# Exploring the conservation of Alzheimer-related pathways between *H. sapiens* and *C. elegans*: a network alignment approach

**DOI:** 10.1038/s41598-021-83892-9

**Published:** 2021-02-25

**Authors:** Avgi E. Apostolakou, Xhuliana K. Sula, Katerina C. Nastou, Georgia I. Nasi, Vassiliki A. Iconomidou

**Affiliations:** 1 Section of Cell Biology and Biophysics, Department of Biology, National and Kapodistrian University of Athens, 15701 Panepistimiopolis, Athens Greece; 2 Present Address: Novo Nordisk Foundation Center for Protein Research, University of Copenhagen, Copenhagen, Denmark

**Keywords:** Computational biology and bioinformatics, Diseases, Neurological disorders, Dementia, Alzheimer's disease

## Abstract

Alzheimer disease (AD) is a neurodegenerative disorder with an –as of yet– unclear etiology and pathogenesis. Research to unveil disease processes underlying AD often relies on the use of neurodegenerative disease model organisms, such as *Caenorhabditis elegans*. This study sought to identify biological pathways implicated in AD that are conserved in *Homo sapiens* and *C. elegans*. Protein–protein interaction networks were assembled for amyloid precursor protein (APP) and Tau in *H. sapiens*—two proteins whose aggregation is a hallmark in AD—and their orthologs APL-1 and PTL-1 for *C. elegans*. Global network alignment was used to compare these networks and determine similar, likely conserved, network regions. This comparison revealed that two prominent pathways, the APP-processing and the Tau-phosphorylation pathways, are highly conserved in both organisms. While the majority of interactions between proteins in those pathways are known to be associated with AD in human, they remain unexamined in *C. elegans*, signifying the need for their further investigation. In this work, we have highlighted conserved interactions related to AD in humans and have identified specific proteins that can act as targets for experimental studies in *C. elegans*, aiming to uncover the underlying mechanisms of AD.

## Introduction

Alzheimer disease (AD) is a chronic, progressive, neurodegenerative disorder, characterized clinically by a gradual impact on mental and cognitive functions, affecting a person’s ability to perform common daily activities^1^. It is the most common cause of dementia and is estimated that by 2050 the number of patients with this disease could exceed 100 million worldwide if no cure is discovered^2^. The pathological hallmarks of AD are amyloid plaques and neurofibrillary tangles; amyloid plaques are created by the extracellular deposition of fibrils consisting of abnormally folded Aβ peptide—a cleavage product of amyloid precursor protein (APP)^3^—while neurofibrillary tangles consist mainly of intracellular hyperphosphorylated twisted filaments of the microtubule-associated Tau protein (Tau hereafter)^1,4^. Moreover, both proteins have the ability to form fibrils extracellularly in vivo*,* and are thus characterized as amyloid fibril proteins by the International Society of Amyloidosis^5^. Therefore, it comes to no surprise that the scientific community has placed much emphasis on understanding the role of these proteins in AD onset.

The study of age-related diseases, such as AD, in humans is practically impossible, due to both the prohibitive nature of an observational study in a human’s lifespan and the serious ethical issues raised on performing experimental studies in humans^6^. Consequently, researchers use model organisms in order to gain insight into the molecular mechanisms underlying these diseases. Animals with close evolutionary relationships to humans, like mouse, pig and non-human primates, can offer great insight for the study of neurodegenerative diseases, and have extensively used as models to study the disease^7–9^. However, in many instances, in vivo experimentation in mammalian model organisms is complex, time- and resource-consuming, therefore other more distant organisms can be used to alleviate these issued. Prominent among them is the nematode *Caenorhabditis elegans,* which is often used as it provides an attractive model for neurodegenerative diseases, due to its distinctive features (see review by Alexander et al.^10^). Most importantly, many human genes (ca. 60–80%) have an ortholog—genes in different species that evolved from a common ancestor gene and possibly retain the same function^11^—in the genome of *C. elegans*^12^. Even though, *C. elegans* has a gene encoding for a protein similar to human Tau (PTL-1)^13^ the APP-related gene (APL-1) does not contain the Aβ peptide sequence. Moreover, *C. elegans* does not possess an ortholog for β-secretase, and thus has no β-secretase activity^10^. For this reason, many transgenic *C. elegans* strains that express human Aβ and Tau sequences in specific cell types have been created^10,13^ and are used as models for the study of AD and other neurodegenerative diseases.

Protein–protein interactions (PPIs) govern most biological processes and investigating them in the context of human diseases is essential to fully comprehend their underlying mechanisms. Networks of PPIs are powerful tools used to conceptualize models of molecular interactions in various biological systems^14,15^. One of the main benefits of PPI networks is that they allow the conversion of a wealth of raw data into reasonably structured visual representations. Nowadays, due to high-throughput techniques, the growth of available PPI data is exponential. Utilization of this data has allowed the study of PPI networks for entire organisms (e.g. *C. elegans*^16^) or for specific diseases(e.g. AD^17^). Moreover, the availability of organism-wide PPI networks has made cross-species network comparisons possible. A popular method for comparing networks is network alignment, which, analogously to genome alignment, aims at mapping the nodes of two or more networks, and thereby, determining topologically and functionally similar regions^18,19^. Conserved network regions can be used to transfer biologically relevant information between humans and model organisms^18,20^. Previous studies^21,22^ have used PPI networks to explore AD mechanisms using the model organism *C. elegans.* To our awareness this is the first effort to employ network alignment for the transfer of knowledge between *C. elegans* and human for the study of this disease.

The goal of this work was the in silico construction and comparison of Alzheimer-related protein–protein interaction networks in humans and *C. elegans*. Our main aim was the discovery of common biological pathways that are conserved in both organisms and are potentially implicated in AD. Study of such pathways will guide experimental studies on the model organism *C. elegans*, and help in the elucidation of mechanisms involved in the pathogenesis of AD.

## Methods

### Protein and interaction datasets

Three network datasets were created for this study, each consisting of a *H. sapiens* PPI network and a *C. elegans* PPI network. Every dataset differs in the method of data collection and the purpose it serves. Cross-species network comparison for each pair of networks in the three datasets was achieved by using one or more network alignment algorithms.

#### APP and Tau network from the amyloid interactome

The first human network we used in this study was isolated from the Amyloid Interactome^23^. The Amyloid Interactome is a PPI network of amyloidogenic proteins and their experimentally verified interaction partners. APP, Tau, their interaction partners, as well as any interactions between them, were extracted from this network. The interactions from the Amyloid Interactome are experimentally validated and extracted from the IntAct database^24^. For the *C. elegans* protein dataset, predicted orthologs of the aforementioned human proteins were recovered using OrthoList2^25^, a compendium of predicted *C. elegans*-human orthologs. The predicted orthologs were further verified through WormBase^26^, a curated database about the genetics, genomics and biology of *C. elegans*. An attempt was initially made to collect interactions between the predicted *C. elegans* orthologs from IntAct, in a manner similar to the Amyloid Interactome, however, only a very small number of such interactions were available. Instead, the STRING database^27^—a database of known and predicted PPIs—was selected, since it integrates data from various sources and covers a vast number of organisms. STRING primarily uses 4 types of evidence—co-expression, experiments, databases and text mining—that it also propagates according to homology; all evidence contribute to a confidence score provided for each PPI. A cutoff interaction score of 0.7 was selected representing interaction partners of high confidence. This dataset was used to evaluate the network alignment algorithms and determine the best parameters that would be used for the subsequent network alignments.

#### APL-1 and PTL-1 network from STRING

Afterwards, the interaction partners of APL-1 and PTL-1—the *C. elegans* orthologs of APP and Tau—were extracted from STRING. A cutoff interaction score of 0.7 was selected representing interaction partners and interactions of high confidence. The predicted human orthologs of those interaction partners were found and constitute the human protein dataset for this pair of networks. For the human network an interaction score of 0.9, representative of the highest confidence level in STRING, was utilized^28^. Furthermore, interactions without experimental validation were filtered out at this stage, in order to increase the reliability of collected data.

#### Top 100 interaction partners for APP and Tau & APL-1 and PTL-1 from STRING

The final dataset is the least biased and therefore was the one primarily used to transfer biological information between the two organisms. The 100 interaction partners of APL-1 and PTL-1 with the best confidence score were collected from STRING, to create the *C. elegans* network. A cutoff interaction score of 0.5 was used for interactions between these proteins, to collect at least 100 additional proteins. Using the same approach, the top 100 interaction partners of APP and Tau were collected from STRING to create the human network. In this case a higher interaction cutoff (0.9), allowed the retrieval of the top 100 interaction partners and the need to reduce it did not emerge. Once again, interactions without experimental validation were filtered out to increase data reliability.

### Network visualization, alignment and comparison

All collected data was visualized as PPI networks using Cytoscape 3.7.2^29^. Cytoscape is a freely available platform for biological network visualization and analysis that provides a vast array of applications for specialized functions. Two such applications used in the current study are the stringApp^30^, which allowed the direct import of networks from the STRING database into Cytoscape and Omics Visualizer^31^, that was used for visualization purposes in this work. Additionally, Cytoscape.js^32^ was used to create interactive networks, available via a web interface at (http://thalis.biol.uoa.gr/celegans_human_AD/), where detailed information about the proteins in the aforementioned networks can be retrieved. Detailed description of the functionalities offered by the web application is available at Supplementary File 1.

Comparison between human and *C. elegans* networks was done using Global Network Alignment (GNA), which aims to locate similarities across entire networks. An array of tools is available to perform GNA, the majority of which are based on a cost function for node similarity and attempt to locate the alignment with a maximum node similarity score^33^. Three algorithms for GNA, namely MAGNA++ ^34^, CytoGEDEVO^35^ and NETAL^36^, were selected and tested to determine the best performing. All three algorithms produce one-to-one node alignments, where each node in one network is uniquely mapped to a single node in the other network.

MAGNA++ uses a genetic algorithm to simulate a population of alignments that evolves in time, until the alignment can no longer be improved. A major advantage of MAGNA++ lies in its effort to maximize edge conservation during the alignment process. CytoGEDEVO is a Cytoscape application that employs the GEDEVO algorithm for network alignment. GEDEVO is based on the graph edit distance between two networks, and attempts to transform one network into the other by applying the minimum number of edge additions and removals. NETAL uses a greedy search algorithm to progressively align node pairs with the best score. An advantage of this tool is that topological information is being renewed during the search for the best alignment.

To optimize each comparison, GNA traditionally uses biological information on top of topological parameters^37^. MAGNA++ and CytoGEDEVO, but not NETAL, accept node similarity inputs allowing for the use of biological information to achieve a better network alignment. Protein sequence similarity was calculated and used as a biological information input, where applicable (Supplementary Table 1). To this end, pairwise sequence alignment was done for every human protein against all *C. elegans* proteins in the dataset using the Needleman-Wunsch algorithm^38^,which aims at finding the best alignment across the entire protein length. The protein sequence similarity was determined as the percentage of aligned residues that matched, i.e. have similar physicochemical properties. Evaluation of the algorithms was done based on their ability to correctly align the nodes of the networks according to their predicted orthologs. Details regarding the evaluation of the algorithms and a flowchart with the step-by-step procedure followed for the creation and alignment of the “*Top 100 interaction partners*” networks are available in Supplementary File 2.

## Results and discussion

In the course of evolution ortholog proteins occasionally maintain the same function across species, as is often reflected by their sequence similarity^39^. Likewise, PPIs between orthologs can be conserved across organisms and these interactions are referred to as interologs. The aim of this work was to explore both of these concepts in the context of comparing homologous PPI networks in *H. sapiens* and *C. elegans* to uncover conserved pathways between these organisms that could be exploited for the study of AD. Three pairs of networks were assembled and compared via GNA (Supplementary Table 5).

### APP and Tau network from the amyloid interactome

**Figure 1:**
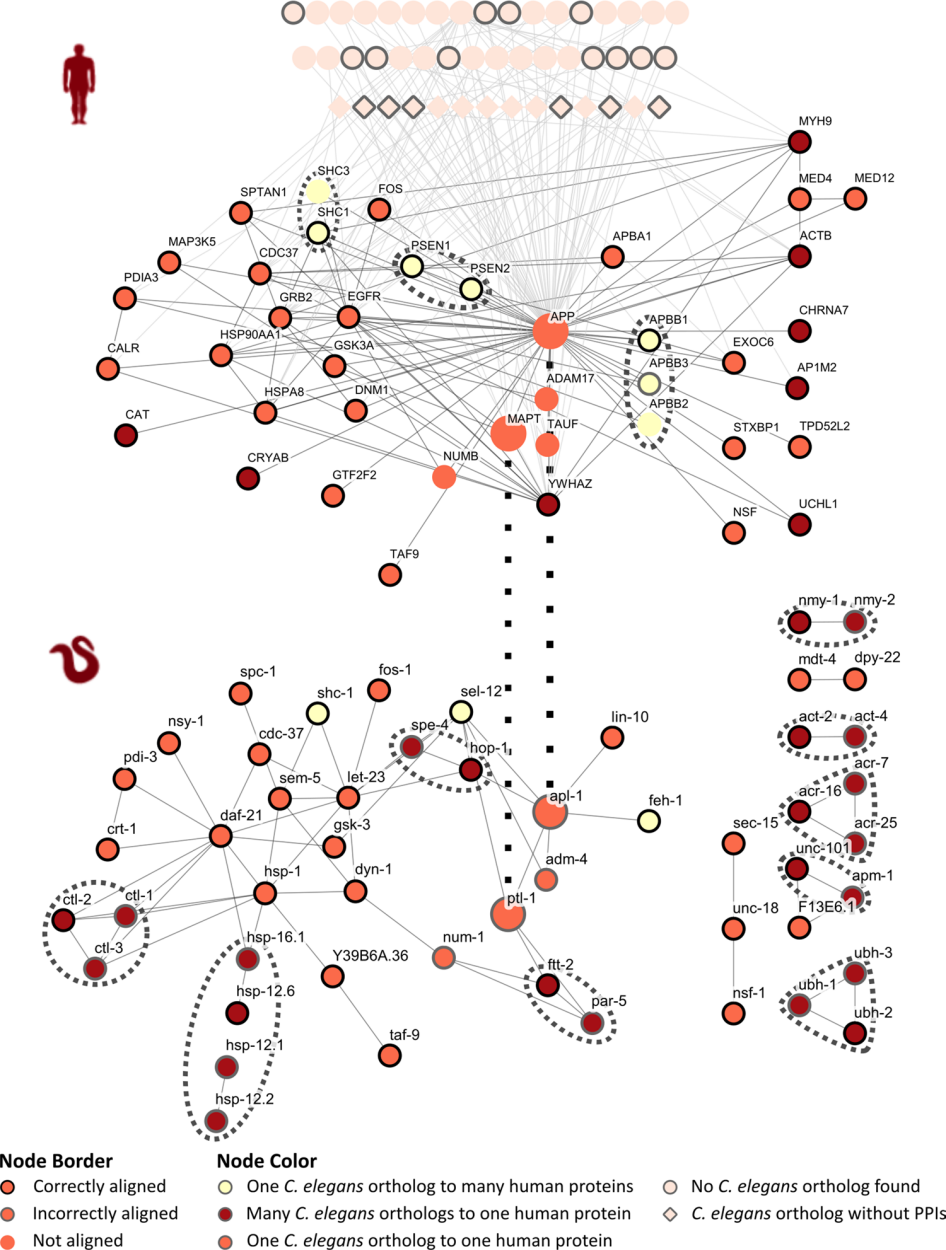
Alignment of human APP and Tau network from Amyloid Interactome and homologous *C. elegans* network by MAGNA++ with combination of biological and topological information, containing both correct and incorrect alignments. The human network (top) and the *C. elegans* network (bottom) are displayed with the layout of nodes corresponding to the predicted ortholog mapping. Human proteins with no predicted orthologs in the *C. elegans* network are isolated on the upper side of the human network. Two dotted black lines show the positioning of ortholog pairs APP & APL-1 and Tau & PTL-1 that were not correctly aligned; APP and Tau were not mapped to any *C. elegans* protein, while APL-1 and PTL-1 were aligned with RNF32 and PRAM1, respectively. All node alignments are available in Supplementary Table 3. Groups of nodes surrounded by dotted lines represent proteins mapped to the same predicted ortholog. These protein nodes have been colored accordingly, to make it easier to detect. Detailed information about the proteins in these networks can be retrieved via the web application (http://thalis.biol.uoa.gr/celegans_human_AD/).

The Amyloid Interactome^23^ reported 86 interaction partners for APP and Tau. This resulted in the human network extracted from it, to consist of 88 proteins and 216 PPIs (Fig. 1). Of the 88 human proteins, 61 were matched to 73 (68 unique) predicted protein orthologs in *C. elegans*; ortholog assignment criteria and mapping can be found in Supplementary File 2 and Supplementary Table 1. A query of IntAct using these 68 proteins returned only 6 interactions amongst the *C. elegans* proteins. Failure to construct a network with data from IntAct, led to the use of STRING instead, and the final *C. elegans* network used in this study consists of 51 proteins and 69 PPIs (Fig. 1).

**Table 1:** GNA algorithm evaluation results. Three GNA algorithms were evaluated based on their ability to correctly align predicted ortholog proteins (51) and identify interologs (22). Bold is used to indicate the best value for each metric. All algorithms failed to align the networks using topological information exclusively and combination with biological information proved necessary for the correct alignment of protein orthologs.

	Alignment based on topological information			Alignment based on topological and biological information	
	*MAGNA*++	*CytoGEDEVO*	*NETAL*	*MAGNA*++	*CytoGEDEVO*
*Correct node alignments*	0	3	0	**33**	24
*Incorrect node alignments*	51	48	51	**18**	27
*Aligned interactions*	56	46	40	31	**39**
*Interologs identified*	0	0	0	**15**	12

The aforementioned pair of networks was used as a “gold-standard” for evaluating the network alignment algorithms. The performance of the GNA algorithms was assessed based on their ability to both align nodes according to the mapping of predicted orthologs and also identify interologs. Manual alignment of the networks according to predicted ortholog mapping revealed the existence of 22 interologs. An endeavor was made to align the networks with the algorithms relying exclusively on network topological information. This resulted in complete failure of the algorithms to align the two networks, with both MAGNA++ and NETAL unable to correctly align any nodes and with CytoGEDEVO making only 3 correct node alignments (Table 1). In an effort to optimize the alignments, biological information was introduced to MAGNA++ and CytoGEDEVO; NETAL on the contrary does not allow the inclusion of such information. The combination of topological and biological information was sufficient to allow the two algorithms to successfully align the two networks, as shown in Table 1. MAGNA++ outperformed CytoGEDEVO, both in correctly aligning predicted ortholog pairs and in identifying interologs (Table 1). More detailed information on the evaluation of the GNA algorithms is available in Supplementary File 2. Ultimately, MAGNA++ correctly aligned 33 out of 51 node pairs according to their predicted ortholog mapping as shown in Fig. 1. Due to some proteins mapping to multiple predicted orthologs, and since GNA algorithms work by aligning unique pairs of proteins, it was inherently impossible for MAGNA++ to achieve a “perfect” alignment. Based on these results, all subsequent network alignments were performed using MAGNA++ with a combination of topological and biological information.

### APL-1 and PTL-1 network from STRING

**Figure 2:**
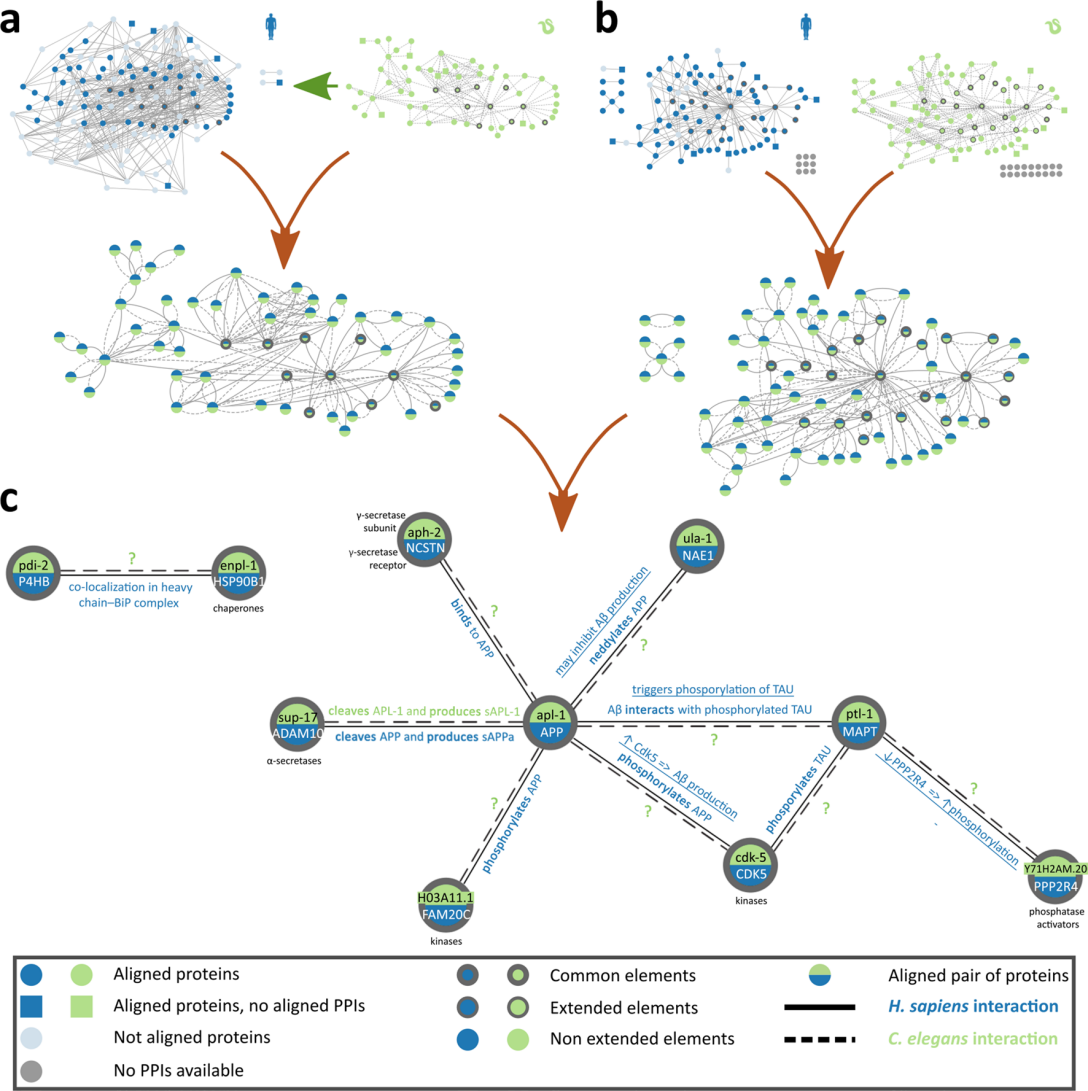
Alignments for the “*APL-1and PTL-1 network from STRING*” and the “*Top 100 interaction partners*” datasets and their common elements. (**a**) The *C. elegans* APL-1 and PTL-1 network from STRING (green) and the homologous human network (blue), with the unified network resulting from their alignment below. (**b**) The Top 100 interaction partners of APL-1 and PTL-1 for the *C. elegans* network (green) and of APP and Tau for the human network (blue). Below these networks is the unified network resulting from their alignment. **(c) **The common elements of the two unified networks (pairs of proteins and their PPIs aligned similarly in both datasets). Omics Visualizer^31^ was used to apply colors on the network’s nodes, with the green half representing the *C. elegans* protein and the blue half representing the human protein. Each half is labeled with their corresponding gene symbol (gene symbol for Tau is MAPT). All human interactions were annotated with at least one supporting publication (Supplementary Table 4); edges with underlined labels indicate annotations directly associated with AD. On the contrary, only one *C. elegans* interaction could be annotated as AD related based on literature evidence.

Next, the *C. elegans* network of APL-1, PTL-1 and their interaction partners was extracted from STRING, using the stringApp^30^, and consisted of 61 proteins and 136 PPIs with experimental evidence (Fig. 2a). In a manner similar to above, the *C. elegans* proteins were mapped to their predicted human orthologs. PPIs were collected from STRING for these proteins, resulting in the human network comprising of 127 proteins and 382 PPIs with experimental evidence (Fig. 2a). Alignment of the aforementioned networks, with the previously established procedure, returned 61 node aligned pairs and a unified network of 55 pairs of *H. sapiens* and *C. elegans* proteins and 92 interologs (Fig. 2a). MAGNA++ succeeded in correctly aligning the majority of predicted orthologs (48 out of 55 pairs), thus providing further validation of its ability to accurately align pairs of networks with orthologous proteins.

### Top 100 interaction partners for APP and Tau & APL-1 and PTL-1 from STRING

The two aforementioned datasets relied on mapping predicted ortholog proteins across the two organisms, a procedure that inevitably introduced bias. Conversely, the “*Top 100 interaction partners*” dataset is the least biased dataset, and was therefore the one primarily used to transfer biological information between the two organisms. The human network had 102 proteins and 164 PPIs, while the *C. elegans* network had 102 proteins and 202 PPIs (Fig. 2b). Because PPIs without experimental validation were excluded, a number of proteins in each network are left with no PPIs. Specifically, interaction data was available for 91 human proteins and 82 *C. elegans* proteins. Alignment of these networks resulted in 82 node aligned pairs and a unified network of 74 aligned protein pairs and 152 conserved interactions (Fig. 2b).

During initial investigation of these networks, the network proteins of both organisms were functionally annotated by searching the literature for information; emphasis was placed on all AD related processes. The vast majority of human proteins in the dataset were found to be AD-associated, thus supporting the relevance of the network in regard to the disease under study. Every association was supported by at least one publication as shown in Supplementary Table 4. Additionally, the evidence provided for the interactions were collected from STRING and the evidence sourced from *C. elegans*, i.e. not transferred from another organism, were extracted (Supplementary Table 2 and Supplementary File 3).

Next, focus was applied to the identification of interactions conserved in both human and *C. elegans*. Common elements between this dataset’s unified network and the unified network of the *“APL-1 and PTL-1 network from STRING”* dataset were extracted. In total 10 pairs of human and *C. elegans* proteins were commonly aligned in both approaches, forming the *Common network* made up of 10 nodes and 9 edges (Fig. 2c). Aside from being commonly aligned, these protein pairs are also predicted orthologs, thereby confirming the conserved interactions between them as interologs. Most of these conserved interactions are linked to post-translational modifications on APP or Tau in human, while information for their role in *C. elegans* was available for only one interaction (Fig. 2c). Limited *C. elegans* evidence were available for 5 of these interactions, the interactions of APL-1 with proteins ULA-1, APH-2 and CDK-5, and the interactions of PTL-1 with CDK-5 and Y71H2AM.20 (Supplementary Table 2 and Supplementary File 3).

### Functional associations: the APP processing pathway

Taking a closer look at the *Common network* in Fig. 2c revealed several interesting associations between the network’s proteins. Located in the *Common network* and interacting with APP are two proteins involved in APP processing, namely disintegrin and metalloproteinase domain-containing protein 10 (ADAM10) and nicastrin (NCSTN). ADAM10 is an α-secretase that proteolytically cleaves APP as part of the non-amyloidogenic APP processing pathway, leading to the release of sAPPα, an important peptide for neuronal function, while also prohibiting the production of Aβ^40^. ADAM10 participates in many important processes, including brain function and development, and is therefore considered a viable drug target; however caution is necessary due to the existence of multiple ADAM10 substrates^41^. Network alignment paired ADAM10 to its ortholog SUP-17, one of two *C. elegans* proteins with α-secretase activity with the other protein being ADM-4. *C. elegans* APL-1, like APP, is cleaved by an α-secretase resulting in the release of sAPL-1^42^. Following α- or β-secretase cleavage, APP is further cleaved by γ-secretase, a complex with four main components: presenilins 1 (PSEN1) and 2 (PSEN2), anterior pharynx-defective-1 (APH1A or APH1B) and nicastrin (NCSTN)^43^. Nicastrin is thought to act as a receptor binding to APP and other γ-secretase substrates^44^. This protein was aligned to its ortholog APH-2, a protein that also participates in the γ-secretase complex of *C. elegans*, functioning similarly to the human nicastrin^45^.

**Figure 3:**
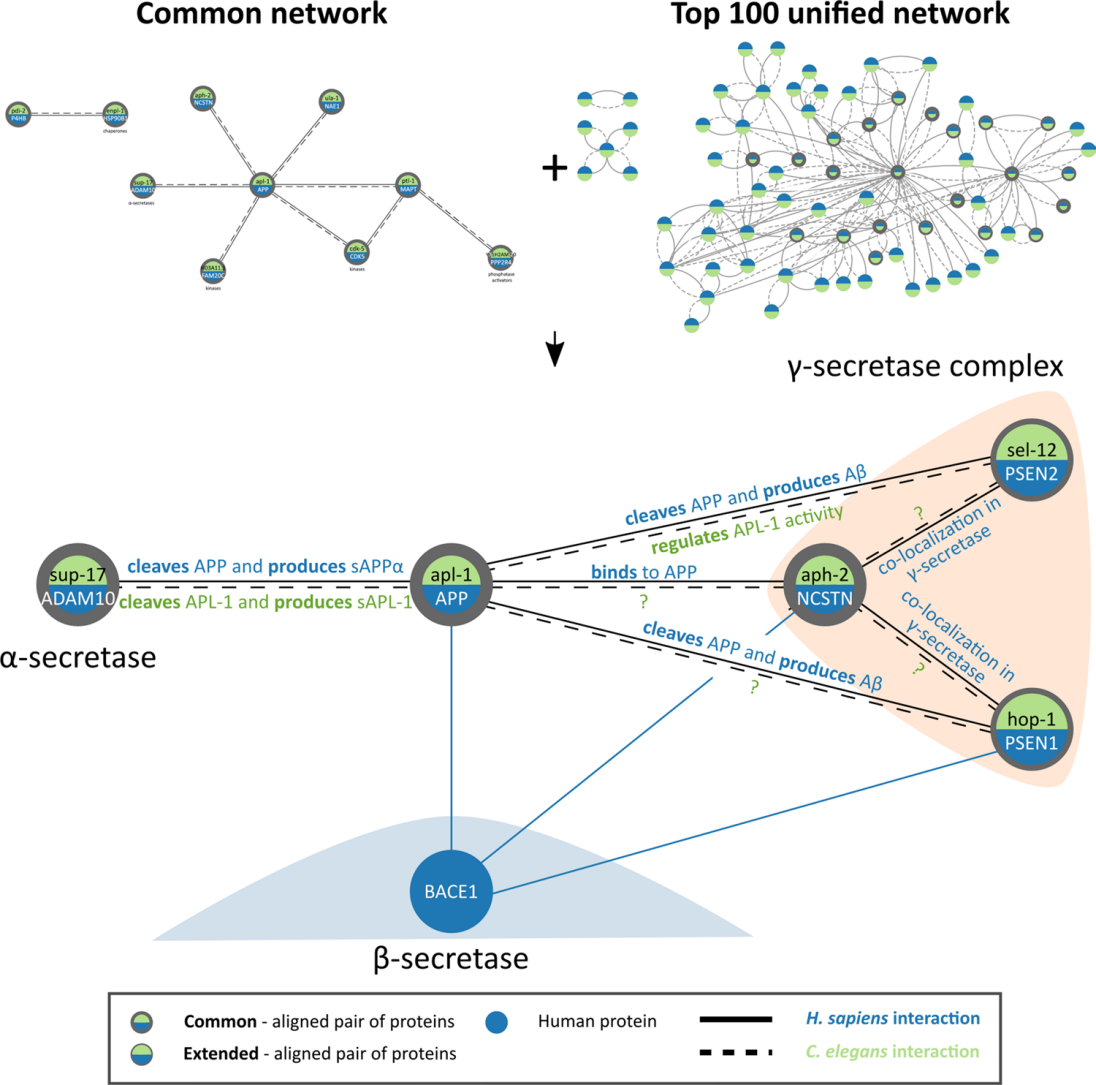
Conservation of the APP processing pathway in *H. sapiens* and *C. elegans*. Shown here are elements of the APP processing pathway found in the *Common Network*, extended with relevant proteins and interactions from the “*Top 100 interaction partners*” dataset. Elements associated with α- and γ-secretase activity are conserved in both organisms, while β-secretase is missing from *C. elegans*. Even though all interactions that take place in this pathway have been experimentally investigated in human, information related to AD could be retrieved only for two *C. elegans* interactions.

Investigation of the network of “*Top 100 interaction partners*” revealed additional elements of the APP processing pathway in the unified network (Fig. 3). These are presenilin 1 (PSEN1) and presenilin 2 (PSEN2) that are responsible for the catalytic activity of the γ-secretase complex^46^. Cleavage of APP by β-secretase followed by γ-secretase, without any α-secretase activity, results in the release of Aβ, according to the amyloidogenic pathway of APP processing^47^. Thus, catalysis by the γ-secretase complex is crucial both for the production of Aβ and the determination of its length and in turn its propensity to aggregation^46^. The catalytic subunits of γ-secretase, PSEN1 and PSEN2, were aligned to *C. elegans* presenilin orthologs HOP-1 and SEL-12, respectively. HOP-1 and SEL-12 are parts of the γ-secretase complex, just like their human counterparts, with evidence suggesting that SEL-12, directly or indirectly, regulates the activity of APL-1^42^. Overall, most of the APP processing machinery, and its corresponding orthologs, arose as important common elements during this work and were determined to indeed be conserved in the model organism. A notable exception is β-secretase (BACE1) that is necessary for the production of Aβ peptide and is absent in *C. elegans*, explaining why Aβ—and not APP—transgenes of this organism are used to model for AD^48^.

For the interaction between APL-1 and SUP-17, as well as for the interactions between APH-2 and proteins SEL-12 and HOP-1, an abundance of evidence was available that was not transferred from homology (Supplementary File 3, Figure S14). On the contrary, most of the evidence for the interactions between APL-1 and proteins SEL-12, HOP-1 and APH-2, are transferred from homology (Supplementary Table 2 and Supplementary File 3, Figure S14). Given the central role of the γ-secretase complex in APP processing in humans, these three proteins are promising targets to experimentally study their interactions between APL-1 and the *C. elegans* γ-secretase complex or to study their role in transgenic *C. elegans*, models that express the human protein APP.

### Functional associations: phosphorylation pathways in AD

**Figure 4:**
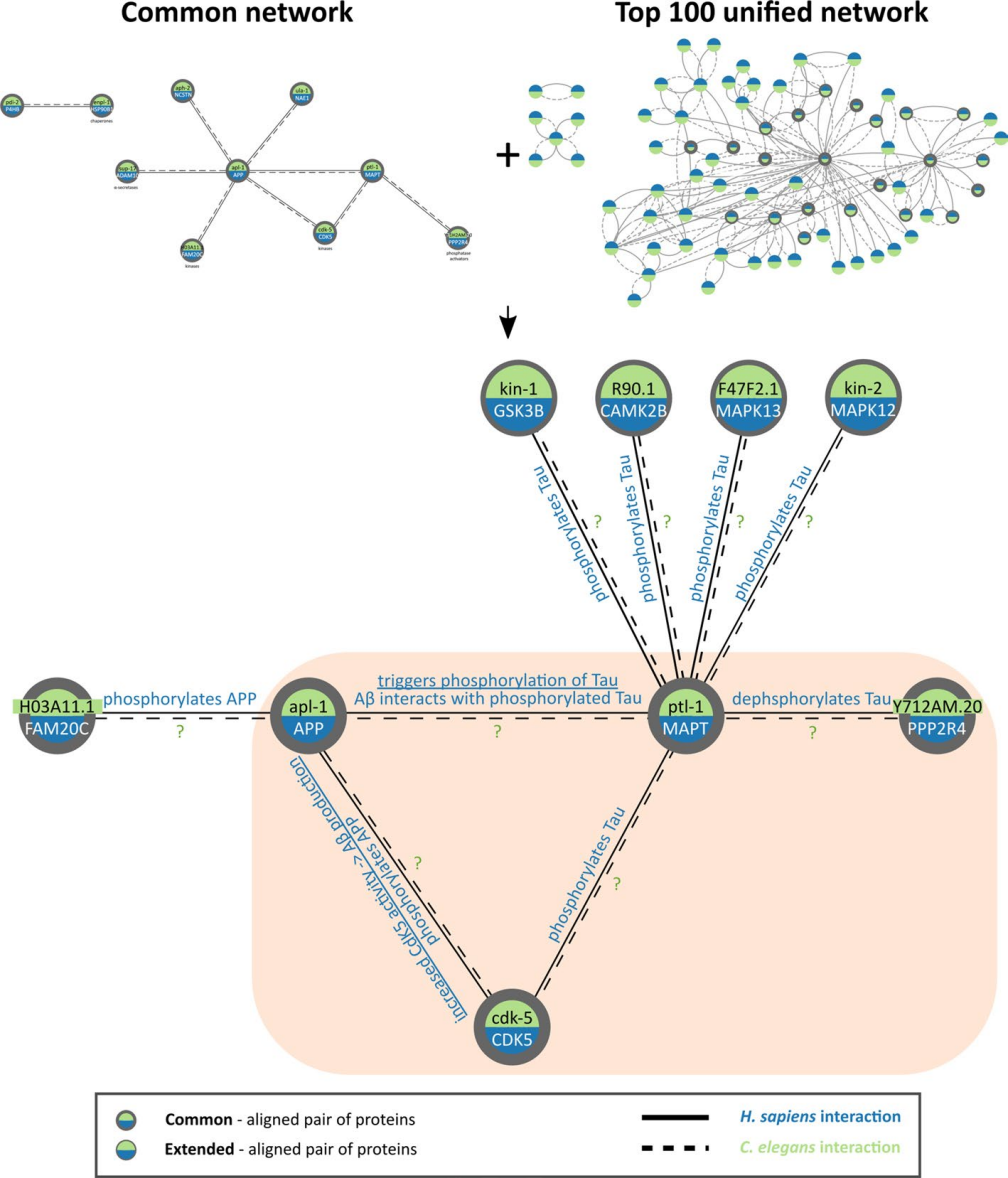
AD associated phosphorylation-related proteins conserved in *H. sapiens* and *C. elegans*. Shown here are elements related to phosphorylation of APP and Tau found in the *Common Network*, extended with relevant proteins and interactions from the “*Top 100 interaction partners*” dataset. The interactions appear to be conserved in both organisms, but no information was available for the *C. elegans* interactions. The gene symbol for Tau is MAPT.

Aside from APP processing, many proteins recovered from the network were involved in phosphorylation (Fig. 4). One of these is cyclin-dependent kinase 5 (CDK5), a prominent kinase in AD, whose aberrant action is associated with pathological processes, including the formation of amyloid plaques and neurofibrillary tangles in AD^49^. CDK5 phosphorylates APP^50^, promoting the production of Aβ peptide^51^, and mediates Tau hyperphosphorylation, leading to its dissociation from microtubules and its aggregation^52^. Furthermore, Aβ peptide was shown to interact with phosphorylated Tau protein in AD brains, potentially causing neuronal damage^53^, while another study reported an affinity of Aβ peptide for non-phosphorylated Tau, triggering its phosphorylation and promoting its aggregation^54^. Modification of Tau by kinases can however be reversed through the action of phosphatases^55^. Primarily responsible for dephosphorylation of Tau is protein phosphatase 2A (PP2A), that is reportedly downregulated in AD, thereby contributing to the hyperphosphorylation of Tau^56^. Located in the *Common network* is the PP2A activator, PPP2R4, which interacts with Tau. In *C. elegans* the predicted orthologs of the aforementioned human proteins also interact, however no additional information is available concerning these interactions. Additionally, a number of kinases that phosphorylate Tau were found in the human network of the “*Top 100 interaction partners*” dataset and were aligned to *C. elegans* kinases. Glycogen synthase kinase-type 3β (GSK3B) is crucial for neurodevelopment and –like CDK5 –it is a main Tau kinase that contributes to the evident hyperphosphorylation of Tau in AD^57^. Another Tau kinase is Ca^2+^/calmodulin (CaM)-dependent protein kinase II (CaMKII)^58^ that is important for synaptic function and is reportedly dysregulated in AD^59^. Lastly, p38 mitogen-activated protein kinases (MAPKs), MAPK12 and MAPK13, were found to interact with Tau; p38 MAPK mediates proinflammatory signaling, is upregulated in AD and is associated with both Aβ and Tau pathology^60^.

Evidence in *C. elegans* was available for the interaction of APL-1 and CDK-5, as well as for the interactions between PTL-1 and proteins CDK-5, F47F2.1, KIN-1, KIN-2 and Y71H2AM.20 was mainly based on homology (Supplementary Table 2L and Supplementary File 3, Figure S15). In the case of APL-1 and CDK-5, considering the central role phosphorylation by CDK5 has in the processing of APP^61^, targeted studies should focus on exploring the phosphorylation potential of the *C. elegans* ortholog or the lack thereof. More specifically, any conserved phosphorylation sites found would help further elucidate the functional role of APP and would be vital to explain the differences in the aggregation mechanisms in the two organisms. Phosphorylation also has a crucial role in the aggregation of Tau and given the relatively recent shift of focus towards Tau-related drug discovery for AD^62^, Tau kinases and phosphatases are subjects of intense study^63^. Despite the importance of Tau, its *C. elegans* ortholog PTL-1 remains understudied with very few available data regarding interactions with other proteins not transferred from homology. This is evident in the results of this study, as only one PTL-1 phosphorylation–related interaction has evidence originated in experiments using *C. elegans*. CDK-5, F47F2.1, KIN-1 and KIN-2 are all kinases aligned to human kinases known to phosphorylate Tau, similarly with Y71H2AM.20 aligned to a human Tau phosphatase. It is therefore possible that these proteins play a role in the phosphorylation of PTL-1 and further studies focusing on the role of this process in *C. elegans* and how it relates to AD-related processes in humans is necessary.

## Conclusions

AD is a complex disease still under study that involves an array of proteins and biological pathways. Two critical players in AD are Aβ and Tau, the main components of amyloid plaques and neurofibrillary tangles, respectively. Unveiling the pathological events leading to the development of AD is a prerequisite for the discovery of the effective prevention and treatment of AD. To this end research often relies in the experimentation on model organisms. In this study we attempted to bridge the gap between AD-related processes in *H. sapiens* and the model organism *C. elegans*. PPI networks were constructed for the human proteins APP and Tau and for their respective *C. elegans* orthologs APL-1 and PTL-1. To achieve this we used network alignment in order to identify proteins and interactions conserved in both organisms that are potentially implicated in AD. Amongst the most prominent pathways, that emerged through this analysis, were the APP processing pathway and the Tau phosphorylation pathway. We extensively reviewed these, critical for AD, pathways and it was revealed that they are highly conserved between the two organisms. Most importantly, we discovered that while the majority of interactions involved in these pathways have been studied in human and associated with AD, almost no information was available about them in *C. elegans*. Additionally, for some of these interactions the evidence in *C. elegans* was very limited indicating the need for more experiments to expand the known *C. elegans* interactome. We therefore managed to showcase significant targets for the study of AD that have been as of yet unexplored in *C. elegans*. In regard to APP processing, candidates for experimental study are the interactions between the members of the *C. elegans* γ-secretase complex, SEL-12, APH-2 and HOP-1, as well as their respective interactions with APL-1. Also, emerging as a target for exploring the neuroprotective role of the human α-secretase responsible for the non-amyloidogenic processing of APP, was its ortholog SUP-17 and its interaction with APL-1. Additionally, several *C. elegans* kinases are prime subjects for investigation, namely, KIN-1, KIN-2, R90.1 and F47F2.1 that interact with PTL-1 and CDK-5, a kinase that interacts with both APL-1 and PTL-1. These as well as other pathways can be further investigated to identify more critical proteins for experimental validation in *C. elegans*. To enable this functionality, we have created a web application that allows detailed browsing of the networks presented in this work (http://thalis.biol.uoa.gr/celegans_human_AD/). In conclusion, we revealed promising AD-related targets for study in *C. elegans* and provided a framework for the transfer of knowledge between *H. sapiens* and *C. elegans* through the computational study of PPI networks.

## Supplementary Information

Supplementary File 1

Supplementary File 2

Supplementary File 3

Supplementary Table 1

Supplementary Table 2

Supplementary Table 3

Supplementary Table 4

## Abbreviations

AD: Alzheimer disease
APP: Amyloid precursor protein
PPIs: Protein-protein interactions
GNA: Global network alignment
