# Supplementary material for: Exploring the conservation of Alzheimer-related pathways between H. sapiens and C. elegans: a network alignment approach
Source: Sci Rep. 2021 Feb 25;11:4572. doi: 10.1038/s41598-021-83892-9 (PMC7907373; doi:10.1038/s41598-021-83892-9)
Supplement: Supplementary file 1 [file 41598_2021_83892_MOESM1_ESM.pdf]

Supplementary File 1
Web Application Guide

Exploring the conservation of Alzheimer-related pathways between *H. sapiens* and *C. elegans*: a network alignment approach

Avgi E. Apostolakou[#], Xhuliana K. Sula[#], Katerina C. Nastou, Georgia I. Nasi and Vassiliki A. Iconomidou*

Section of Cell Biology and Biophysics, Department of Biology, National and Kapodistrian University of Athens, Panepistimiopolis, Athens 15701, Greece

*To whom correspondence should be addressed

[#]Equally contributing authors

Associate Prof. Vassiliki A. Iconomidou

Section of Cell Biology and Biophysics, Department of Biology,

National and Kapodistrian University of Athens, Panepistimiopolis,

Athens 15701, Greece

Phone: +30 210 727 4871

Fax: +30 210 727-4254

e-mail: veconom@biol.uoa.gr

http://biophysics.biol.uoa.gr

Web application

Cytoscape.js, a JavaScript graph library, was used to create an interface for interactive exploration of the *H. sapiens*, *C. elegans* and aligned networks. Details about these networks can be found in the main manuscript. Following are some instruction on how to navigate through these networks.

Selecting a network

A drop-down menu is available on the left side for selecting the network to view. Three (3) networks are available for each dataset, those being the respective *H. sapiens* network, the *C. elegans* network and the aligned network. The aligned network is the result of network alignment with MAGNA++ and the optimized parameters.

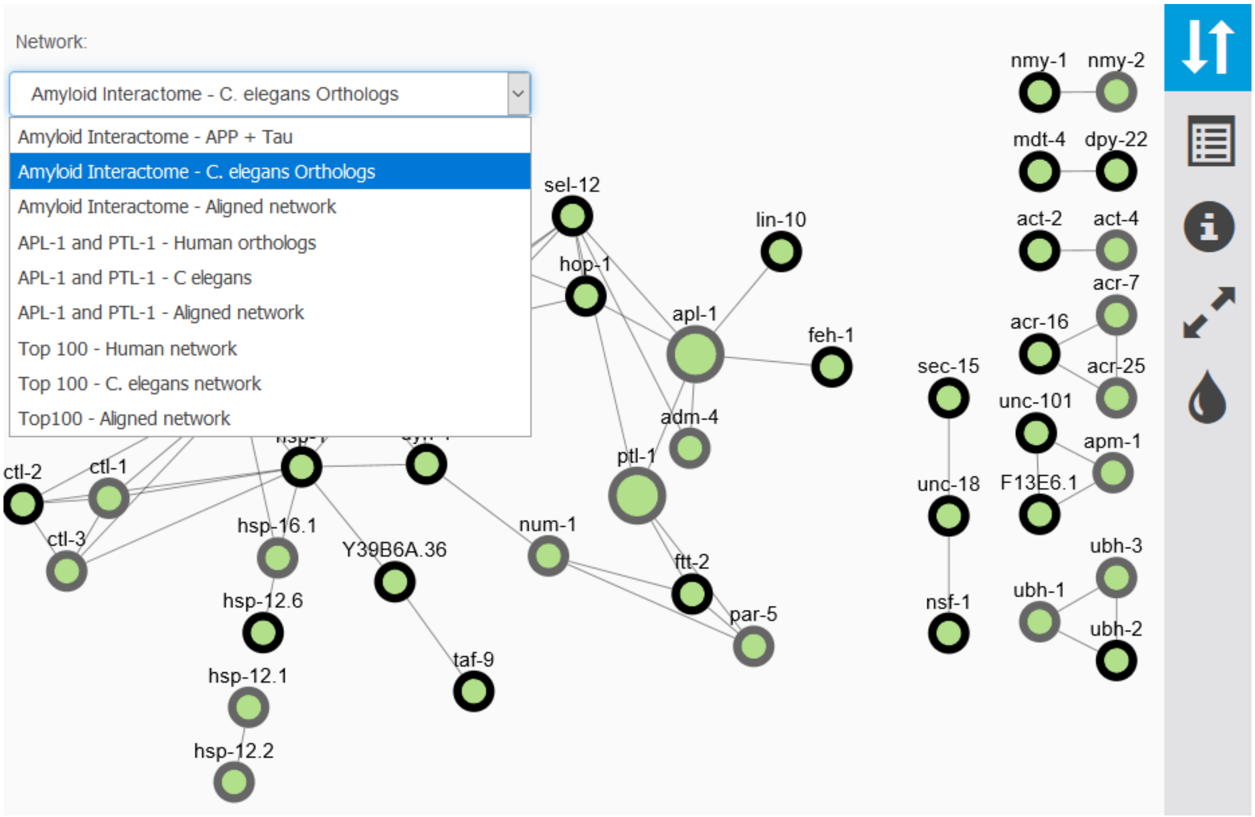

Toolbar options

On the right side is a bar with 5 buttons:

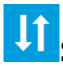Show or hide the Toolbar

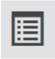 Show or hide a Table with information about the selected node(s)

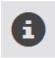 Show or hide the Network Selection menu

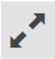Fit network to view

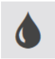Set a different Background color

Table with node information

In the Table information is displayed about the selected node(s). To select a node Left click on it and to move it (Click & Drag). If the network on display is a *H. sapiens* or *C. elegans* network, the node represents a protein and the Table contains basic information about it including its **gene name**, its corresponding **STRING identifier** and **description**. Otherwise, in the case of an aligned network, the **STRING identifiers** and **gene names** are given for the aligned pair of proteins.

SUID	id	shared_name	stringdb_species	stringdb_description
196052	196052	9606.ENSP00000284981	Homo sapiens	Amyloid beta (A4) precursor protein; N-APP binds TNFRSF21 triggering caspase activation and degeneration of both neuronal cell bodies (via caspase-3) and axons (via caspase-6); Endogenous ligands

Available networks

APP and Tau network from the Amyloid Interactome dataset

Nodes that were correctly aligned have a black border, those incorrectly aligned have a grey border and nodes without any border were not aligned (only present in the Human network).

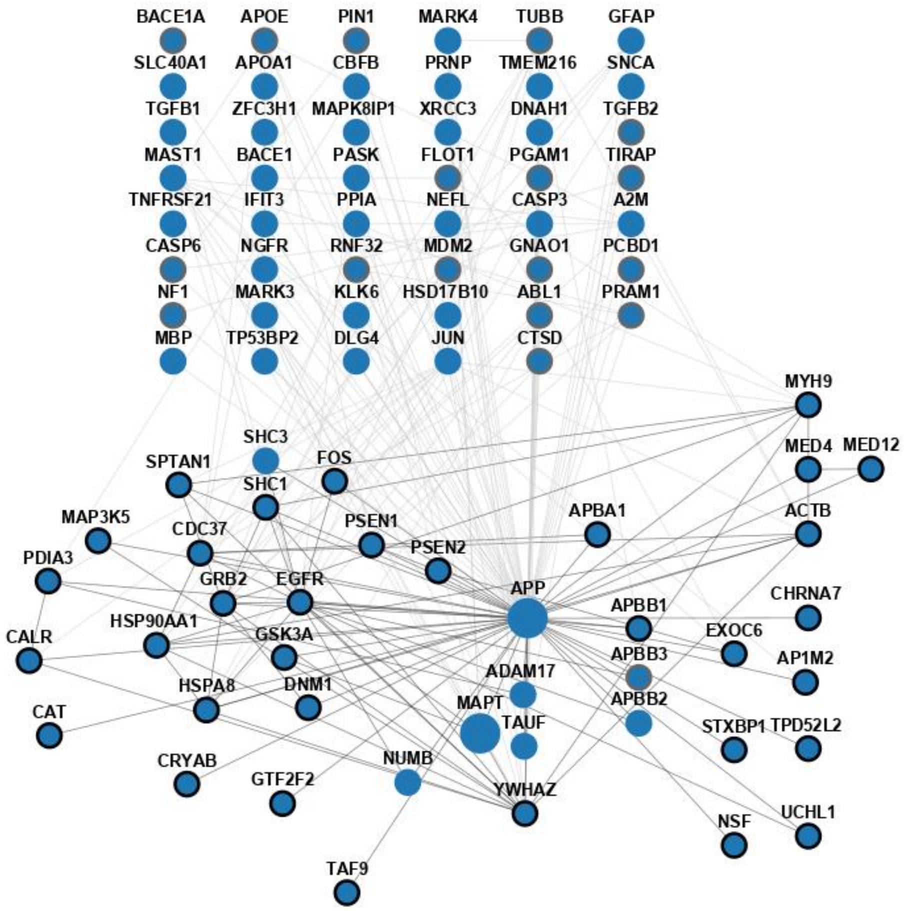

Figure S1. The Human APP and Tau network from the Amyloid Interactome.

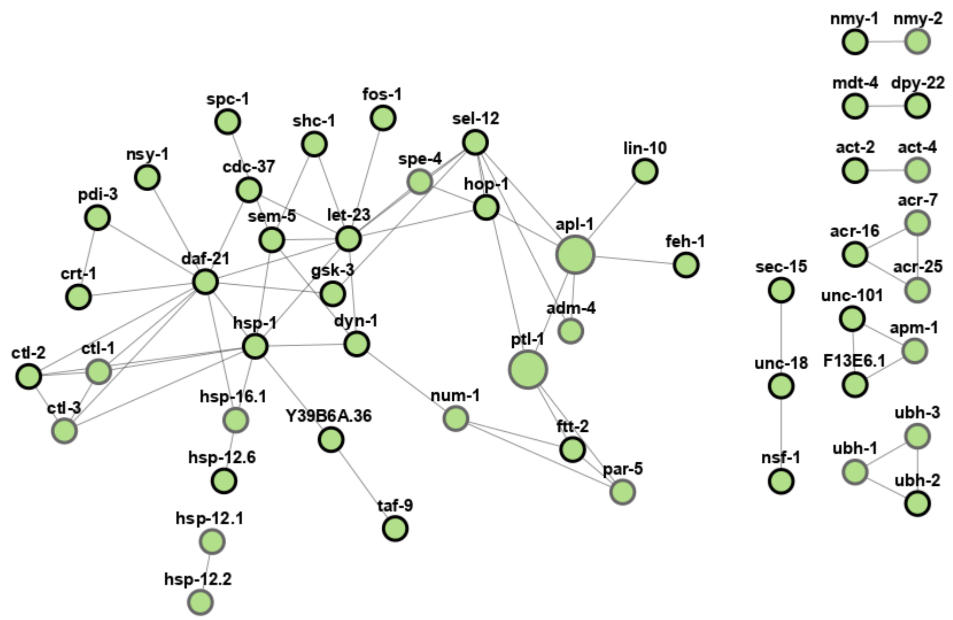

Figure S2. The orthologous *C. elegans* network.

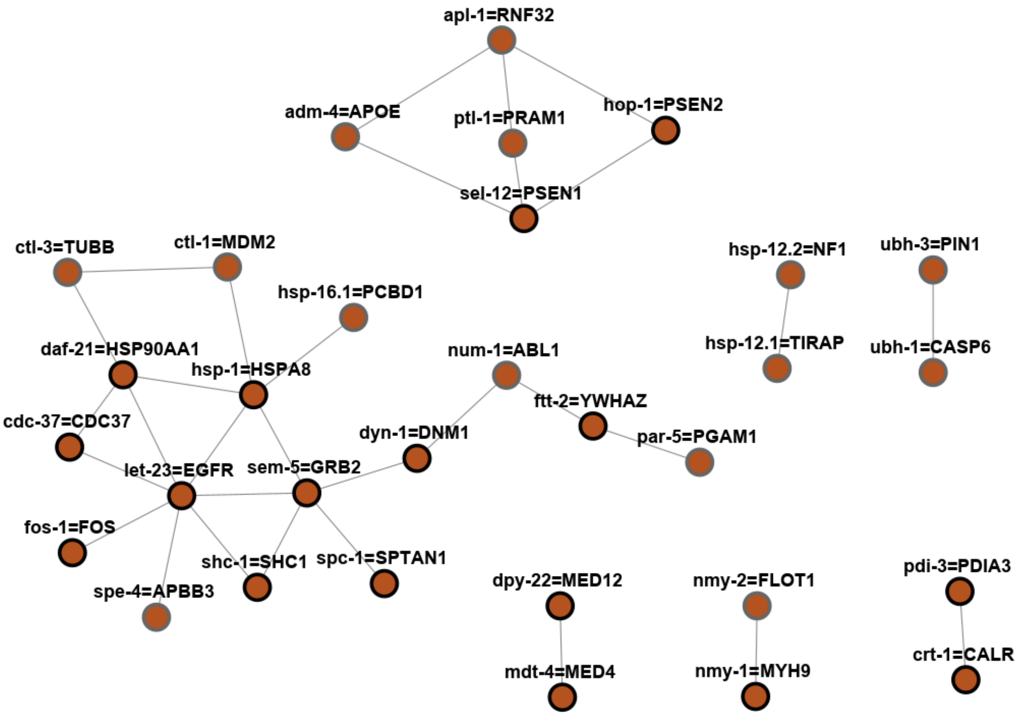

Figure S3. The Aligned network between the networks shown in Figures S1 and S2.

APL-1 and PTL-1 network from STRING dataset

Nodes corresponding to proteins located in the *Common network* are marked with a black border.

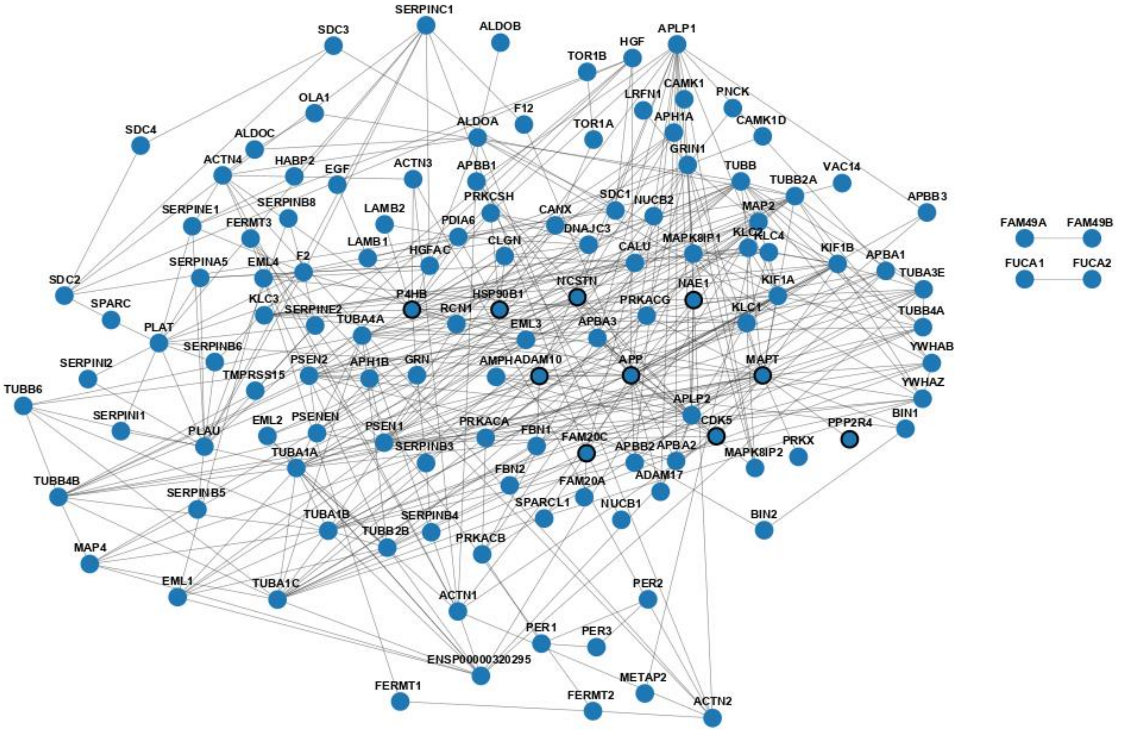

Figure S4. The Human orthologous network to the APL-1 and PTL-1 network from STRING.

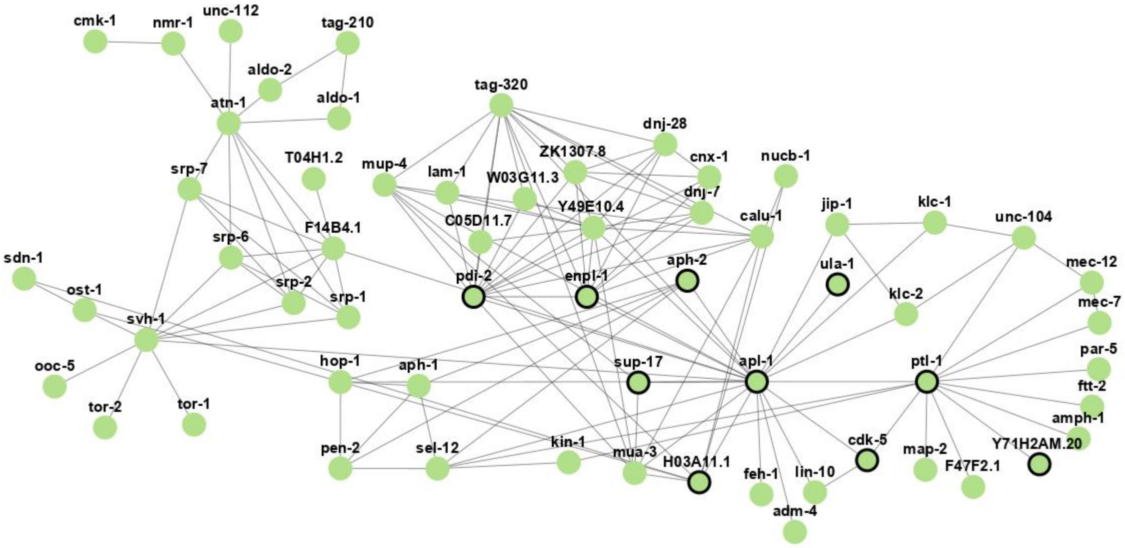

Figure S5. The *C. elegans* APL-1 and PTL-1 network from STRING.

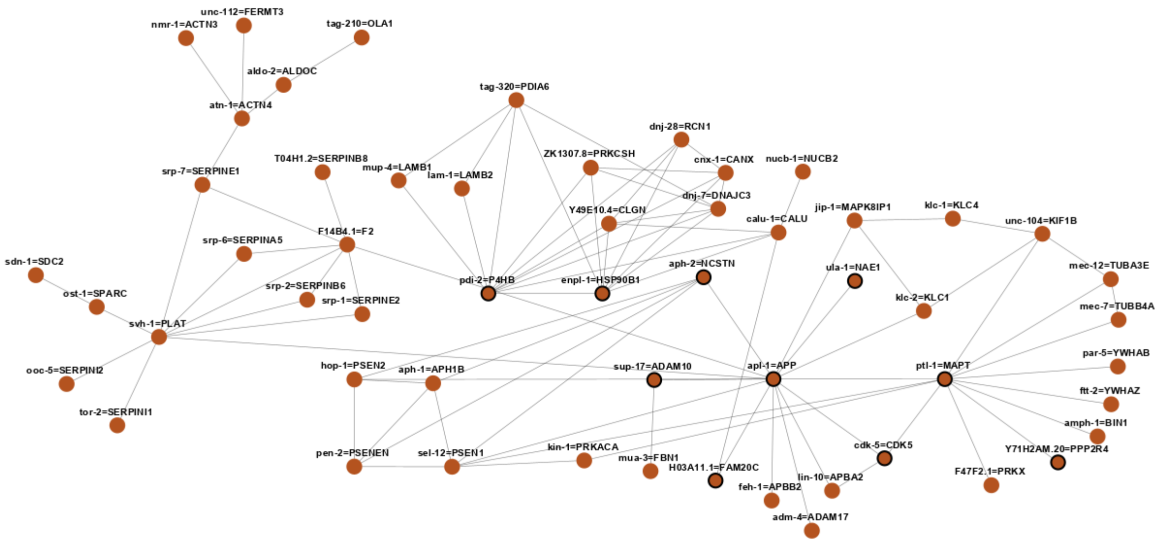

Figure S6. The Aligned network between the networks shown in Figures S4 and S5.

Top 100 interaction partners for APP and Tau & APL-1 and PTL-1 from STRING dataset

Nodes corresponding to proteins located in the *Common network* are marked with a black border

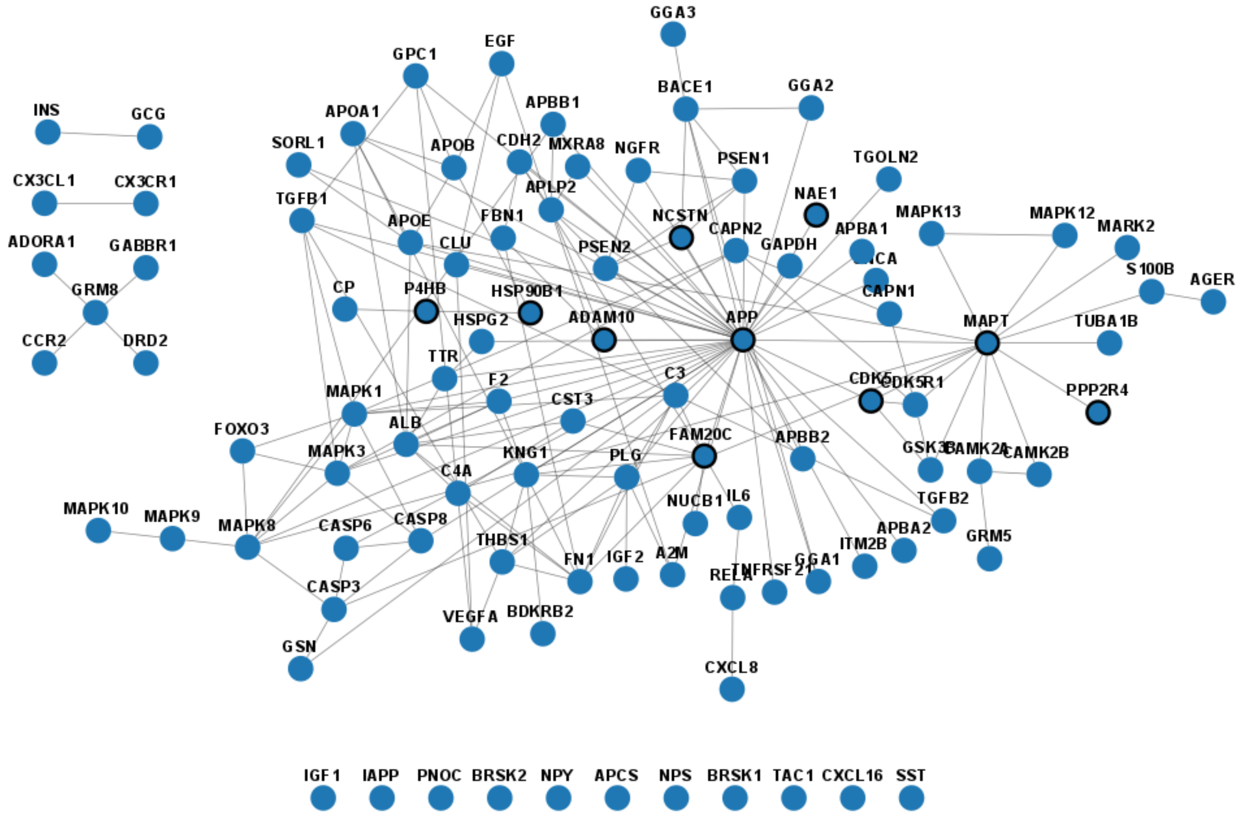

Figure S7. The Top 100 interaction partners for APP and Tau network (*H. sapiens*).

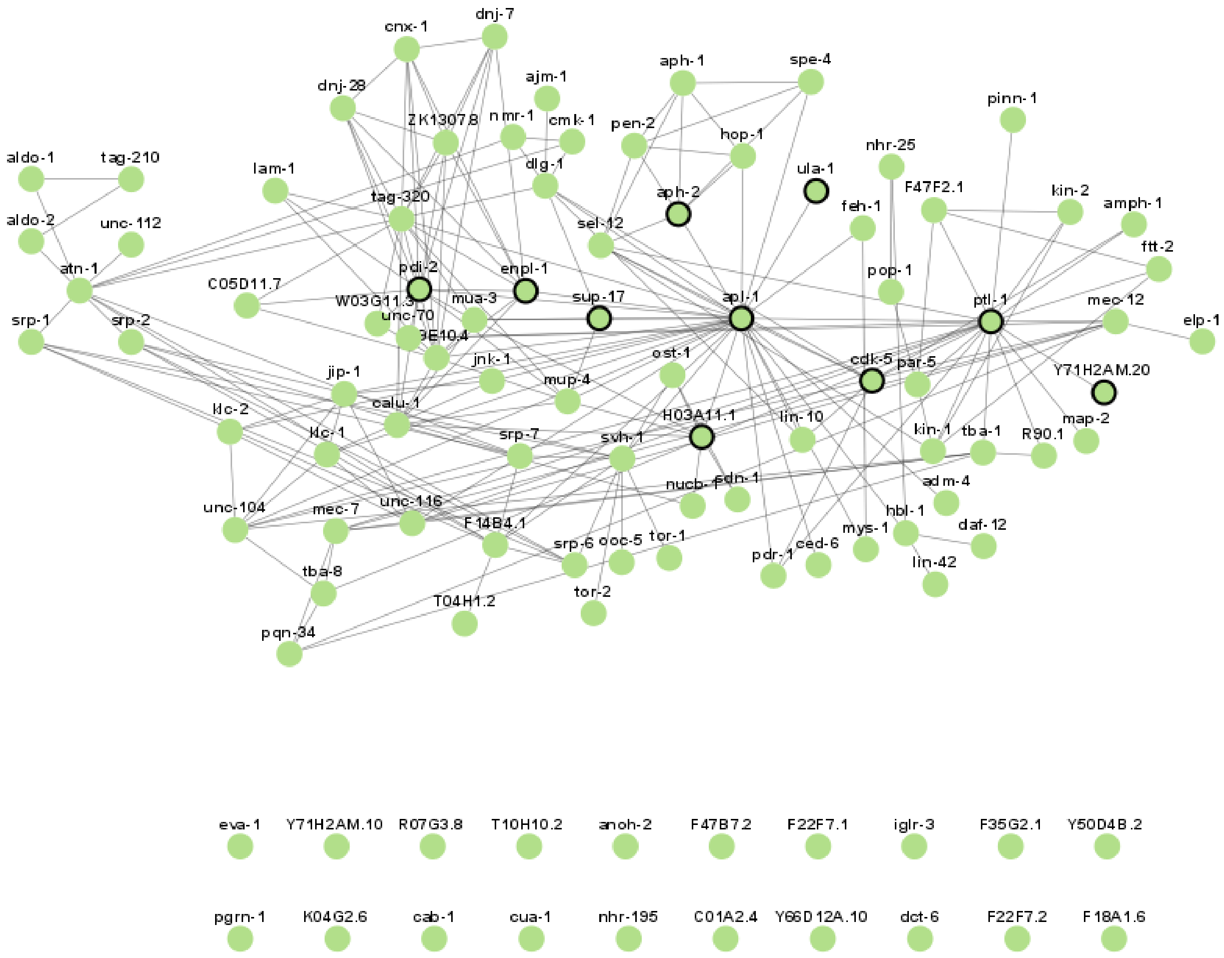

Figure S8. The Top 100 interaction partners for APL-1 and PTL-1 network (*C. elegans*).

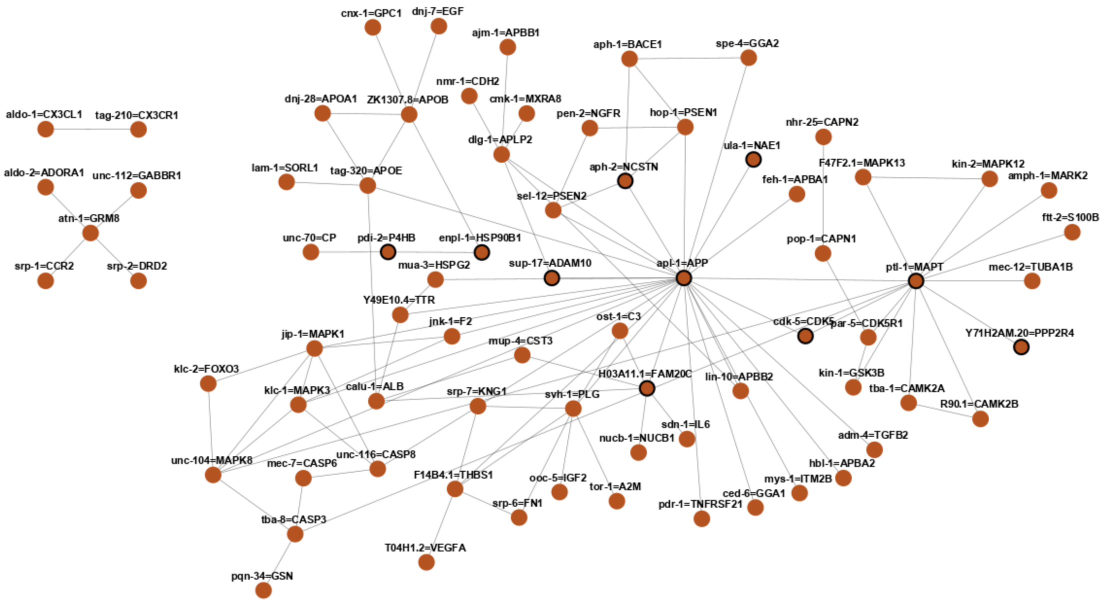

Figure S9. The top-100 interaction partners aligned network.
