# Supplementary material for: Exploring the conservation of Alzheimer-related pathways between H. sapiens and C. elegans: a network alignment approach
Source: Sci Rep. 2021 Feb 25;11:4572. doi: 10.1038/s41598-021-83892-9 (PMC7907373; doi:10.1038/s41598-021-83892-9)
Supplement: Supplementary file 2 [file 41598_2021_83892_MOESM2_ESM.pdf]

Supplementary File 2
Additional Methods

Exploring the conservation of Alzheimer-related pathways between *H. sapiens* and *C. elegans*: a network alignment approach

Avgi E. Apostolakou[#], Xhuliana K. Sula[#], Katerina C. Nastou, Georgia I. Nasi and Vassiliki A. Iconomidou*

Section of Cell Biology and Biophysics, Department of Biology, National and Kapodistrian University of Athens, Panepistimiopolis, Athens 15701, Greece

*To whom correspondence should be addressed

[#]Equally contributing authors

Associate Prof. Vassiliki A. Iconomidou

Section of Cell Biology and Biophysics, Department of Biology,

National and Kapodistrian University of Athens, Panepistimiopolis,

Athens 15701, Greece

Phone: +30 210 727 4871

Fax: +30 210 727-4254

e-mail: veconom@biol.uoa.gr

http://biophysics.biol.uoa.gr

Visual summary of the methodology

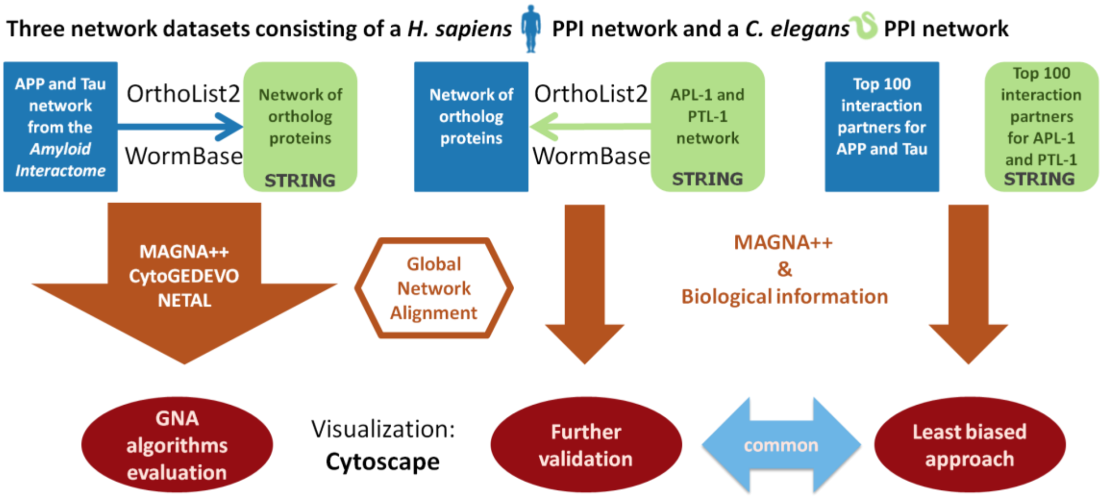

Figure S10. Depicted above are the steps taken to produce the results of this work. The three datasets consisting of a *H. sapiens* (blue) and a *C. elegans* (green) network are shown, with an arrow corresponding to the direction of ortholog mapping. The first network dataset was aligned using three GNA algorithms, therefore allowing for their evaluation. Alignment of the remaining networks was accomplished with the best performing GNA algorithm in combination with biological information. While the second dataset provided further validation for the alignment protocol, the final dataset represented the least biased approach (not reliant on ortholog mapping). Emphasis was placed in the proteins commonly aligned in the latter two datasets. Finally, all networks were visualized using Cytoscape.

Flowchart depicting the methodology used for the "*Top 100 interaction partners*" dataset

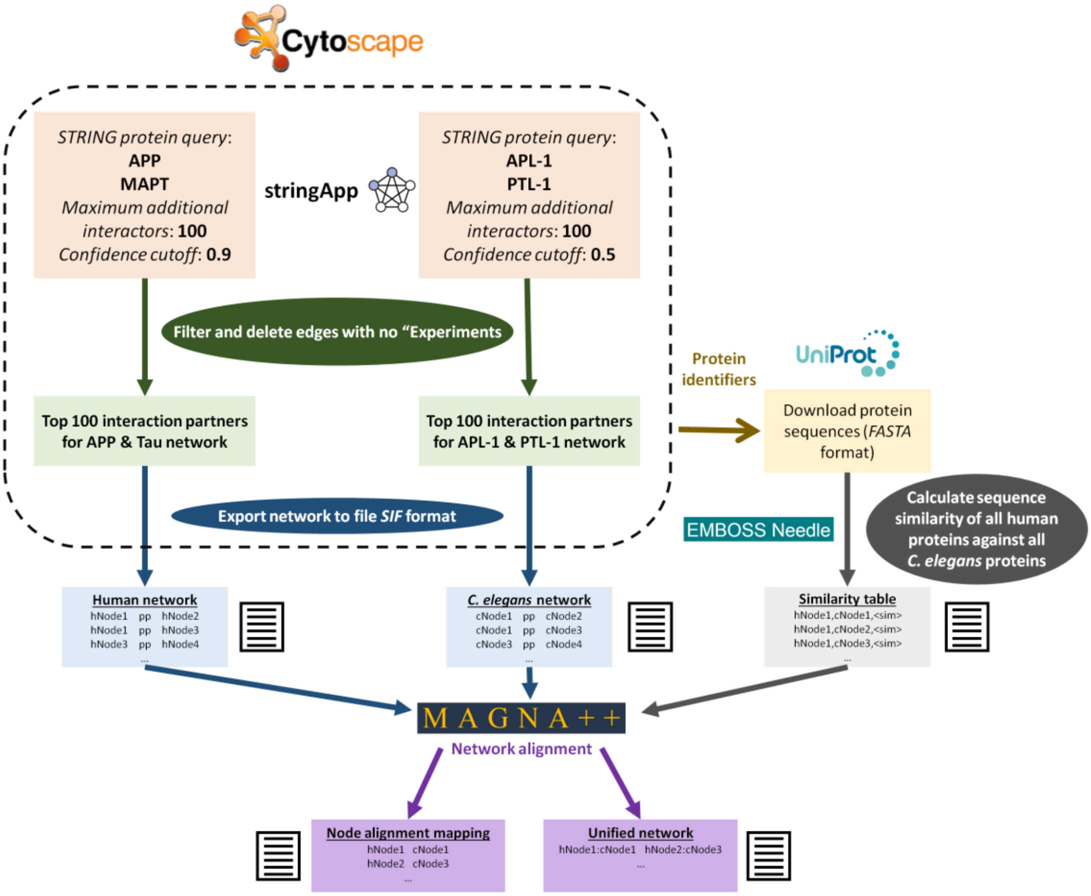

Figure S11. Depicted above are the steps taken to create and align the "*Top 100 interaction partners*" networks. Processes performed in Cytoscape are placed within the dashed-line frame. First the networks for human and *C. elegans* are, independently, imported from STRING directly into Cytoscape via the stringApp. Next all interactions not supported by experimental evidence (edges with no value for attribute "Experiments") were selected via Filter and removed. The networks were then exported to two files respectively for human and *C. elegans*, these files are in a SIF format that can be used as input for the network alignment algorithm MAGNA++. Additionally, the protein identifiers for the nodes of each network were extracted and the corresponding sequences were downloaded using UniProtKB in a FASTA format. These sequences were used to calculate the sequence similarity of all possible pairs of human and *C. elegans* proteins of this dataset with the help of the tool EMBOSS Needle that performs global pairwise sequence alignment. A comma separated file was created containing the sequence similarity of each pair and used as input for node similarity by MAGNA++. The aforementioned three files were input for MAGNA++, which after finishing the alignment returned two files used, one with the node alignment mapping and one with the unified network.

Criteria for selecting orthologs

To determine orthologs we used **OrthoList2** [1] and **WormBase** [2] for verification. **OrthoList2** provides the consensus of 6 databases, with **HomoloGene** [3] considered the most reliable and **OrthoMCL** [4] and **Ensembl Compara** [5] the least specialized. WormBase is a curated database for nematodes and was therefore considered as the most reliable source of information.

Steps followed for ortholog selection:

- Genes not reported in **WormBase** as orthologs were removed
- In **UniProtKB** [6] there are 5 types of evidence for the existence of a protein:
    1. *Experimental evidence at protein level*
    2. *Experimental evidence at transcript level*
    3. *Protein inferred from homology*
    4. *Protein predicted*
    5. *Protein uncertain*

  Proteins with experimental evidence at either protein or transcript level were accepted, while proteins inferred from homology were accepted only if **WormBase** reported them as primary orthologs.
- In case of multiple potential orthologs:
    - Orthologs predicted by 2 or fewer databases were rejected
    - Only orthologs reported by **WormBase** were accepted
- Legacy orthologs were accepted only after literature search and confirmation by **WormBase**

GNA algorithm parameterization

1) The **NETAL** [7] algorithm was used via the web interface: http://bioinf.modares.ac.ir/software/netal/

The parameters used were the *Similarity and interaction Score* – **0.01** and the *Iterations* – **2.** This algorithm does not permit the introduction of biological information.

2) The **GEDEVO** algorithm was used via the Cytoscape application **CytoGEDEVO** [8].

The recommended parameters were used for the alignment relying only on topological information. For the incorporation of biological information, the node similarity file was used as input and the parameters used were *Data model* – **Similarity** and *Value Range* – **Clamp**.

3) In order to use **MAGNA++** [9] we downloaded the source code from https://www3.nd.edu/~cone/MAGNA++/downloads.html and used the *MAGNA++ Command Line Interface (CLI).*

The recommended values for the following parameters were used:

- *Measure of edge conservation* – $\mathbf{S^3}$
- *Population size* – **15000**
- *Number of generations* – **2000**

When biological information is used in **MAGNA++** the parameter α determines how strongly the alignment will be affected by the biological similarity versus the edge conservation. A value of **1** means only biological information will be taken into account and a value of **0** means that only edge conservation will be used. The values **0.4** and **0.6** were tested to allow for an equal contribution of biological and topological determinants. Ultimately the value **0.4** was selected as it led to the correct alignment of *33* orthologs, contrary to **0.6** which led to the correct alignment of only *21* pairs.

GNA algorithms evaluation

Initial attempts at aligning the two networks using exclusively topological information resulted in complete failure (Table S1). We therefore decided to introduce biological information to be combined with topological information; this was possible only with **MAGNA++** (Table S2) and **CytoGEDEVO** (Table S3). Two common measures of protein similarity were used, namely Sequence Identity and Sequence similarity. The values for these properties were calculated by performing Pairwise sequence alignment of the human proteins against the *C. elegans* proteins. The **Needleman-Wunsch algorithm** [10] was used since it employs dynamic programming to return the optimal global alignment quickly; the tool **needle** from the **EMBOSS suite** [11] was used for this purpose. Additionally, in order to determine how well the networks could be aligned if the pairs of orthologs were taken into account by the algorithms, we used a third binary measure whose value was **1** for every pair of orthologs and **0** for every pair of non-orthologs.

To evaluate the performance of the *GNA algorithms* we used the number of *correct node alignments* and the number of *interologs identified*. An aligned pair of nodes was considered correct only if it corresponds to ortholog proteins and incorrect otherwise. An *interolog* is a conserved interaction, i.e. when two human proteins as well as their orthologs interact. Manual alignment of the networks according to ortholog mapping revealed *22 interologs*.

Table S1. Results for alignment based on Topological Information for all tested algorithms

	MAGNA++	***CytoGEDEVO***	***NETAL***
Correct node alignments	0	3	0
Incorrect node alignments	51	48	51
Aligned interactions	56	46	40
Interologs identified	0	0	0

Table S2. Results for alignment by MAGNA++ with different biological information

	No biological information	***Sequence Identity***	***Sequence similarity***	***Values (1 for ortholog pair, 0 otherwise)***
Correct node alignments	0	28	33	37
Incorrect node alignments	51	23	18	14
Aligned interactions	56	35	31	36
Interologs identified	0	11	15	20

Table S3. Results for alignment by CytoGEDEVO with different biological information

	No biological information	***Sequence Identity***	***Sequence similarity***	***Values 1 for ortholog pair, 0 otherwise***
Correct node alignments	3	19	24	37
Incorrect node alignments	48	32	27	14
Aligned interactions	46	43	39	33
Interologs identified	0	7	12	17

References

1 Kim, W., Underwood, R. S., Greenwald, I. & Shaye, D. D. OrthoList 2: A New Comparative Genomic Analysis of Human and Caenorhabditis elegans Genes. *Genetics* **210**, 445-461, doi:10.1534/genetics.118.301307 (2018).

2 Lee, R. Y. N. *et al.* WormBase 2017: molting into a new stage. *Nucleic Acids Res* **46**, D869-D874, doi:10.1093/nar/gkx998 (2018).

3 Sayers, E. W. *et al.* Database resources of the National Center for Biotechnology Information. *Nucleic Acids Res* **48**, D9-D16, doi:10.1093/nar/gkz899 (2020).

4 Li, L., Stoeckert, C. J., Jr. & Roos, D. S. OrthoMCL: identification of ortholog groups for eukaryotic genomes. *Genome Research* **13**, 2178-2189, doi:10.1101/gr.1224503 (2003).

5 Vilella, A. J. *et al.* EnsemblCompara GeneTrees: Complete, duplication-aware phylogenetic trees in vertebrates. *Genome Research* **19**, 327-335, doi:10.1101/gr.073585.107 (2009).

6 The UniProt Consortium. UniProt: a worldwide hub of protein knowledge. *Nucleic Acids Res* **47**, D506-D515, doi:10.1093/nar/gky1049 (2019).

7 Neyshabur, B., Khadem, A., Hashemifar, S. & Arab, S. S. NETAL: a new graph-based method for global alignment of protein-protein interaction networks. *Bioinformatics* **29**, 1654-1662, doi:10.1093/bioinformatics/btt202 (2013).

8 Malek, M., Ibragimov, R., Albrecht, M. & Baumbach, J. CytoGEDEVO-global alignment of biological networks with Cytoscape. *Bioinformatics* **32**, 1259-1261, doi:10.1093/bioinformatics/btv732 (2016).

9 Vijayan, V., Saraph, V. & Milenkovic, T. MAGNA++: Maximizing Accuracy in Global Network Alignment via both node and edge conservation. *Bioinformatics* **31**, 2409-2411, doi:10.1093/bioinformatics/btv161 (2015).

10 Needleman, S. B. & Wunsch, C. D. A general method applicable to the search for similarities in the amino acid sequence of two proteins. *J Mol Biol* **48**, 443-453, doi:10.1016/0022-2836(70)90057-4 (1970).

11 Rice, P., Longden, I. & Bleasby, A. EMBOSS: the European Molecular Biology Open Software Suite. *Trends Genet* **16**, 276-277, doi:10.1016/s0168-9525(00)02024-2 (2000).
