# Supplementary material for: Exploring the conservation of Alzheimer-related pathways between H. sapiens and C. elegans: a network alignment approach
Source: Sci Rep. 2021 Feb 25;11:4572. doi: 10.1038/s41598-021-83892-9 (PMC7907373; doi:10.1038/s41598-021-83892-9)
Supplement: Supplementary file 3 [file 41598_2021_83892_MOESM3_ESM.pdf]

Supplementary File 3
Scored networks

Exploring the conservation of Alzheimer-related pathways between *H. sapiens* and *C. elegans*: a network alignment approach

Avgi E. Apostolakou[#], Xhuliana K. Sula[#], Katerina C. Nastou, Georgia I. Nasi and Vassiliki A. Iconomidou*

Section of Cell Biology and Biophysics, Department of Biology, National and Kapodistrian University of Athens, Panepistimiopolis, Athens 15701, Greece

*To whom correspondence should be addressed

[#]Equally contributing authors

Associate Prof. Vassiliki A. Iconomidou

Section of Cell Biology and Biophysics, Department of Biology,

National and Kapodistrian University of Athens, Panepistimiopolis,

Athens 15701, Greece

Phone: +30 210 727 4871

Fax: +30 210 727-4254

e-mail: veconom@biol.uoa.gr

http://biophysics.biol.uoa.gr

Interaction confidence score

STRING, the database used to gather protein interaction data in this work, uses primarily 4 types of evidence – co-expression, experiments, databases and text mining – that it also propagates according to homology; all evidence contribute to a confidence score provided for each PPI. In addition to the calculated score for each interaction, STRING allows the user to download all interaction data for a single organism, in this case *C. elegans,* including the scores for every type of evidence from 4 different sources. This evidence is split into those directly regarding the organism in question and those resulting by homology transfer from other organisms.

Using these values, we managed to calculate new confidence scores for the interactions in the "Top 100 interactors network" – using the same scoring scheme that STRING uses[1] (https://string-db.org/help/faq/) – for interactions that are derived exclusively from *C. elegans*. The network consists of 202 protein interactions and we discovered that 159 have a score higher than 0.15 when using exclusively *C. elegans* evidence, which is the cutoff used to indicate interactions of low confidence by STRING (by default this score includes also evidence from homology transfer). In fact, 137 of these interactions had a newly calculated score higher than 0.4 corresponding to interactions of medium confidence, which is the default cutoff when retrieving results from STRING. All detailed scores are available in Supplementary Table 3.

[1] von Mering, C., et al. ***STRING: known and predicted protein-protein associations, integrated and transferred across organisms.*** *Nucleic Acids Res 33, D433-437, doi:10.1093/nar/gki005 (2005).*

Figures of *"Top 100 interaction partners" C. elegans* networks with scores computed for *C. elegans* data only (no homology transfer)

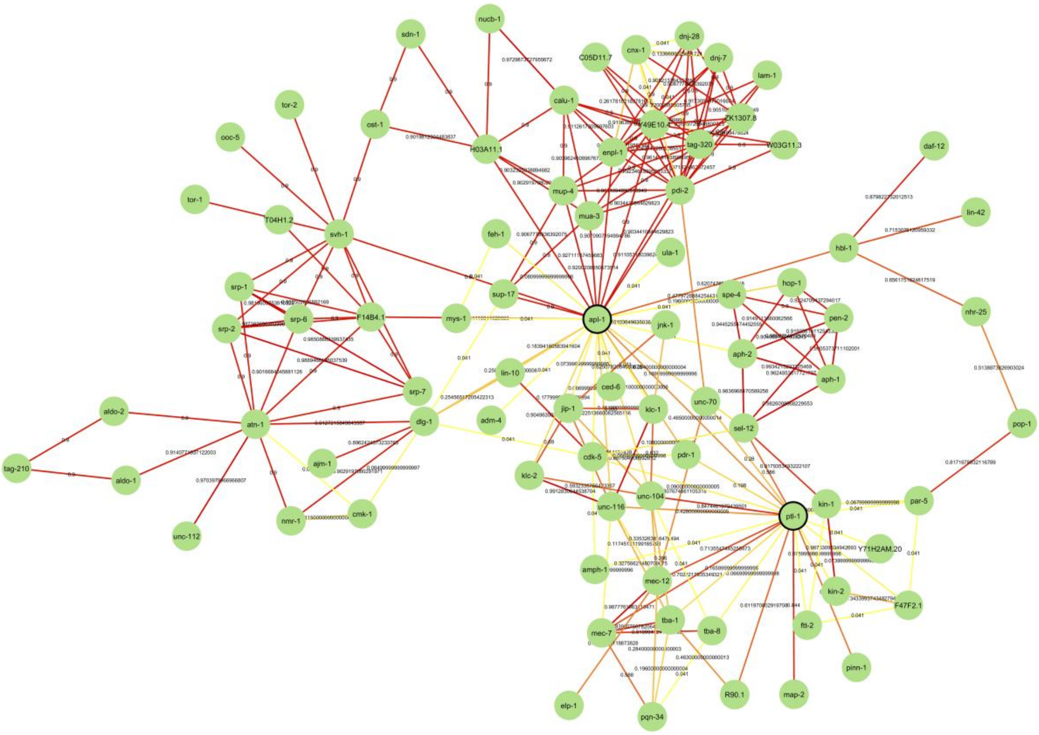

Figure S12. The "*Top 100 interaction partners" C. elegans* network. Edges are labeled with the score and colored according to their score (higher score – red to lower score – yellow). The network is also available via the web interface at http://thalis.biol.uoa.gr/celegans_human_AD/

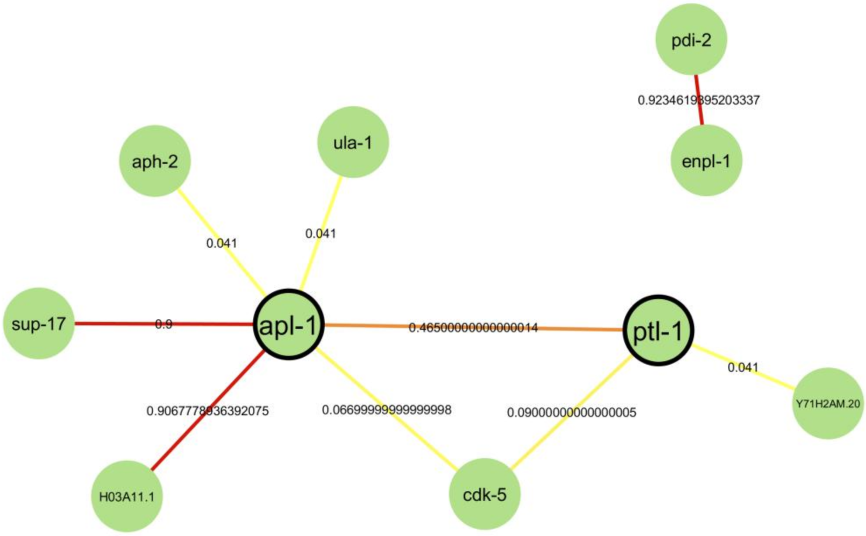

Figure S13. The common elements of the two unified networks from the "*APL-1and PTL-1 network from STRING*" and the "*Top 100 interaction partners*" datasets (pairs of proteins and their PPIs aligned similarly in both datasets). Edges are labeled with the score and colored according to their score (higher score – red to lower score – yellow).

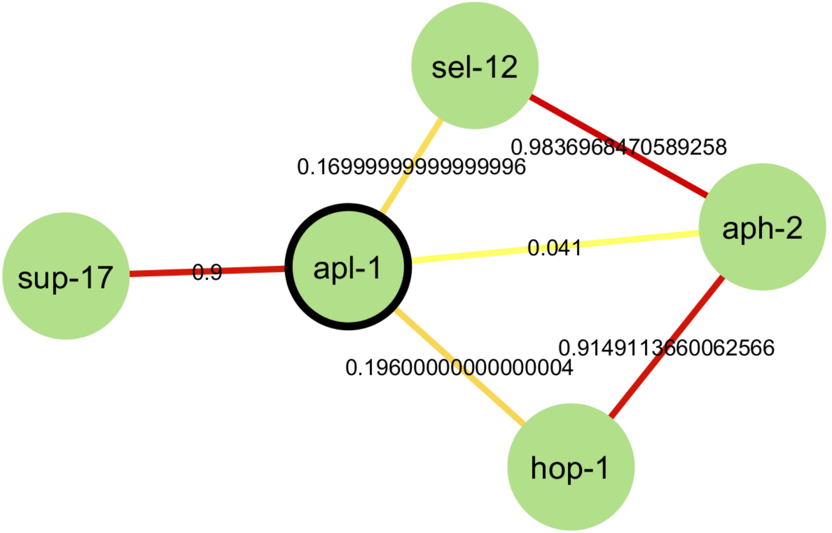

Figure S14. The APP processing pathway found in the "*Top 100 interaction partners" C. elegans* network. Edges are labeled with the score and colored according to their score (higher score – red to lower score – yellow).

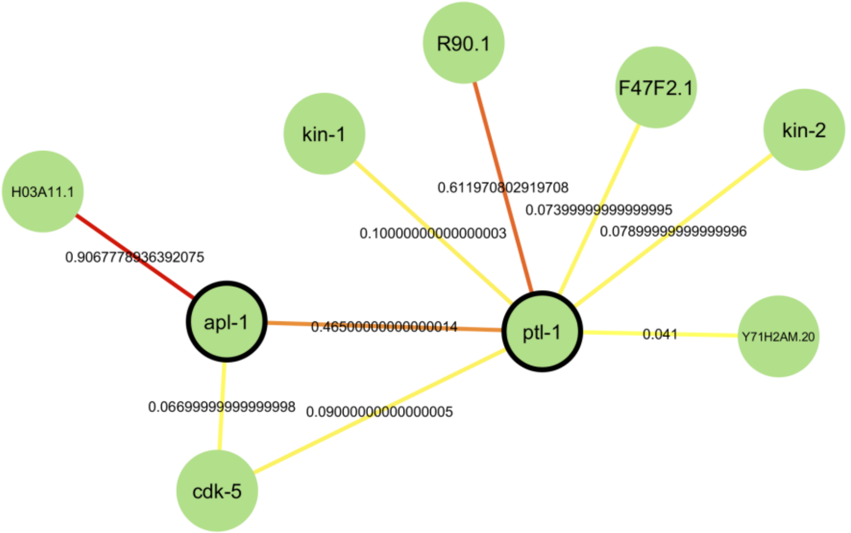

Figure S15. The Tau phosphorylation pathway found in the "*Top 100 interaction partners" C. elegans* network. Edges are labeled with the score and colored according to their score (higher score – red to lower score – yellow).
